# Expression of epithelial growth factors and of apoptosis-regulating proteins, and presence of CD57+ cells in the development of inflammatory periapical lesions

**DOI:** 10.1590/1678-7757-2021-0413

**Published:** 2022-02-21

**Authors:** Walter Arthur Silva Valente, Déborah Barrocas, Luciana Armada, Fábio Ramôa Pires

**Affiliations:** 1 Universidade Estácio de Sá Programa de Pós-Graduação em Odontologia Rio de Janeiro RJ Brasil Universidade Estácio de Sá, Programa de Pós-Graduação em Odontologia, Rio de Janeiro, RJ, Brasil.

**Keywords:** Bcl2, CD57, EGF, KGF, Ki-67, Periapical lesions

## Abstract

The mechanisms that stimulate the proliferation of epithelial cells in inflammatory periapical lesions are not completely understood and the literature suggests that changes in the balance between apoptosis and immunity regulation appear to influence this process.

**Objective:**

To evaluate the expression of the epidermal growth factor (EGF), its receptor (EGFR) and of the keratinocyte growth factor (KGF), the presence of CD57+ cells, the epithelial cell proliferation index, and the expression of the Bcl-2 protein in inflammatory periapical lesions (IPL) at different stages of development.

**Methodology:**

Our sample was composed of 52 IPLs (22 periapical granulomas - PG - and 30 periapical cysts - PC), divided into three groups: PGs, small PCs, and large PCs. Specimens were processed for histopathologic and immunohistochemical analyses. Sections were evaluated according to the amount of positive staining for each antibody.

**Results:**

We found no significant differences among the groups regarding Bcl-2 (p=0.328) and Ki-67 (p>0.05) expression or the presence of CD57+ cells (p=0.748). EGF (p=0.0001) and KGF (p=0.0001) expression was more frequent in PCs than in PGs, and CD57+ cells were more frequent in IPLs with intense inflammatory infiltrates (p=0.0001). We found no significant differences in KGF (p=0.423), Bcl-2 (p=0.943), and EGF (p=0.53) expression in relation to inflammatory infiltrates or to the type of PC epithelial lining, but observed greater KGF expression (p=0.0001) in initial PCs. EGFR expression was similar among the groups (p>0.05).

**Conclusion:**

More frequent EGF and KGF expression in PCs and the greater presence of CD57+ cells in lesions with intense inflammatory infiltrates suggest that these factors influence IPL development. The greater KGF expression in initial PCs suggests its importance for the initial stages of PC formation.

## Introduction

Infections in the root canal system can cause a group of inflammatory conditions known as inflammatory periradicular lesions (IPLs). The most common IPLs are periapical granulomas (PG) and periapical cysts (PC); both appear as unilocular radiolucent lesions with well-defined borders associated with necrotic teeth.^1,2^ The histological characterization of PGs involve connective tissue infiltrated by a predominantlty chronic inflammatory infiltrate, small vessels, and, sometimes, proliferating epithelial islands that still fail to produce an inner cavity.^1-3^ In turn, PCs consist of cystic cavities lined by stratified squamous epithelia of irregular thickness showing espongiosis and containing a fibrous capsule of connective tissue which holds the same components found in PG.^1-3^ The literature claims PCs originate from pre-existing PGs; however, we still lack the full understanding of the exact mechanisms modulating this progression.^3^

The literature shows that, in response to an inflammatory stimulus, the proliferation of quiescent epithelial cell rests of Malassez within the periodontal ligament originate the non-keratinized stratified squamous epithelium that lines PCs.^3^ Various phenomena stimulating the proliferation of epithelial cells appear to influence this process, such as the higher expression of growth factors and their receptors, and changes in the balance between apoptosis and immunity regulation.^4-8^ The CD57 protein relates to cytotoxic activity and mediate immunosuppression,^9^ whereas Bcl-2, to cell division regulation, inhibiting apoptosis in several cell types.^10^ The epidermal growth factor (EGF) stimulates the proliferation of keratinocytes and fibroblasts, and relates to cell proliferation and wound healing.^5^ Its receptor, EGFR, is found in epithelial cells with proliferative potential.^4^ The keratinocyte growth factor (KGF) also correlates with epithelial cell growth and differentiation, which inflammatory stimuli influence.^11^

The literature has previously shown the individual importance of these cell types, growth factors (and their receptors), and anti-apoptotic proteins for IPLs.^4-8^ However, no studies have compared the expression of these factors in IPLs at their different stages of development. Thus, this study aimed to evaluate the expression of epithelial growth factors and of proteins regulating apoptosis and immune responses, as well as cell proliferation markers in PGs and PCs at different stages of IPL development. We hypothesize that the expression of these markers varies across these stages.

## Methodology

Files from the Oral Pathology Laboratory at the Universidade Estácio de Sá, in Rio de Janeiro, Brazil were reviewed between 2013 and 2014. In total, 52 IPLs (22 PGs and 30 PCs) were selected for this retrospective study. All specimens analyzed came from surgical procedures recommended for reasons unrelated to this study, and were stored in flasks containing 10% buffered formalin solutions. Specimens were subjected to conventional histological processing and embedded in paraffin wax.

The clinical (lesion site, and clinical signs and symptoms), demographical (patients’ age and gender), and radiographical information (lesion size and border outline) of all specimens were obtained from laboratory files. Specimens from patients with immunological disorders such as diabetes, HIV infection, and autoimmune diseases were excluded, as were cases in which the surgical specimens were insufficient for adequate histological analyses or obtained by incisional biopsy.

The IPLs were divided into three groups: PGs (22 cases, 42%), small PCs (nine cases, 17% - radiographical diameter smaller than 10 mm) and large PCs (21 cases, 41% - diameter greater than 10 mm); the last two groups were classified based on the greater diameter observed in high-quality periapical radiographs.

Paraffin blocks containing the selected specimens were cut into 5 µm-thick sections, stained with hematoxylin and eosin, and observed under an optical microscope (Leica DM500, Heerbrugg, Switzerland). Histological analyses were conducted by two previously calibrated examiners according to the following parameters: type of inflammatory infiltrate (mixed or chronic); intensity of the inflammatory infiltrate (focal/slight or moderate/intense); and presence of clefts from cholesterol crystals. For PCs, the thickness of the epithelial lining was also analyzed (atrophic or hyperplastic).

Immunohistochemical reactions were carried out according to a previously described standardized protocol,^12-15^ using 3 µm-thick sections obtained from the paraffin blocks. Sections were placed on silanized slides, deparaffinized in xylene, and hydrated by soaking in decreasing ethanol concentrations. Antigen retrieval was performed in 10 mM citrate buffer solutions (pH 6) in a microwave oven. Endogenous peroxidase was blocked with 3% hydrogen peroxide, followed by incubation of the slides with anti-EGF (ABCAM AB9695, polyclonal, 1:500), anti-EGFR (Novocastra, clone 384, 1:500), anti-KGF (ABCAM AB90259, polyclonal, 1:1000), anti-Bcl-2 (Biocare Medical CM003C, clone 100/05, 1:200), anti-CD57 (Biocare Medical CM007C, clone NK-1, 1:100), and anti-Ki-67 (Dako M7240, clone MIB-1, 1:100) primary antibodies diluted in antibody diluent solutions (Dako). Dilutions followed manufacturers’ instructions and laboratory protocols. The slides were incubated in a moist chamber for 16 hours at 4°C. According to the manufacturers’ instructions, tissues known to react to each antibody served as positive controls, whereas these primary antibodies were omitted for the negative controls. After incubation with the primary antibodies, slides were washed in PBS and incubated with a secondary antibody (LSAB, Dako) for 30 minutes at 37°C, washed again in PBS, and incubated with a streptavidin-biotin-peroxidase complex (LSAB, Dako) for 30 minutes at 37°C. The reaction was revealed with a 3,3’-diaminobenzidine tetrachloride solution (DAB, Dako), followed by counterstaining with Carazzi’s hematoxylin and mounting on Entellan (Merck, Germany).

The immunoslides were observed under an optical microscope, and the most representative area (“hot spot”) for each slide stained for CD57, KGF, EGF, and EGFR was chosen. The percentage of positive cells in the total amount of cells in the area was estimated by 400x magnification (high-power view) under light microscopy. CD57, EGF, EGFR, and KGF expression was also scored as negative/focal (0-5%) or positive (greater than 5%). Cells were identified according to protein-specific marker positivity or negativity. Bcl-2 expression was scored as negative/focal (0-5%) or positive (greater than 5%) due to the presence of both cellular and extracellular immunoexpression. For anti-Ki-67 antibodies, the number of ‘positive’ epithelial cell nuclei in each specimen was estimated out of the total number of epithelial cell nuclei present within two fields observed under high magnification (1000x). Therefore, the mean proliferation index per field observed under high magnification was obtained for each specimen. The two fields selected for observation under high magnification were Ki-67 expression “hot spots”. All slides were analyzed by two previously calibrated examiners. In case of disagreement, the final decision was based on a group discussion. The methods used for the histological and immunohistochemical analyses have been validated and previously used in other studies performed by the same group.^12-15^

Clinical, demographic, radiographic, histological, and immunohistochemical data were recorded and analyzed descriptively and comparatively via the SPSS software (Statistical Program for Social Sciences, version 20, IBM, USA). Differences in EGF, EGFR, KGF, Bcl-2, CD57, and Ki-67 expression among the groups were analyzed using the T-test and the Pearson’s chi-squared test with p<0.05 (5% significance level). This study was approved by the Ethics Committee at the Universidade Estácio de Sá (No. 1.242.574), and conducted in full accordance with the ethical principles of the Declaration of Helsinki (2008).

## Results

The IPLs included in this study affected 32 women (62%) with a mean age of 44.9 years, and 20 men (38%) with a mean age of 41.9 years, with no significant differences in age and gender distribution among the groups (Table 1). Symptoms were present in 36% of the IPLs, without differences among the three groups, but local swelling was more common in small PCs (Table 1). The mean diameter of the IPLs was 14.4 mm, based on the largest dimension observed radiographically, and we considered most case images as well-defined, with no group differences (Table 1).

**Table 1:** Distribution of the clinical and radiological parameters by group (PG - periapical granuloma; PC - periapical cyst)

Clinical parameter	Group				P-value
	PG	Small PC	Large PC	Total	
	Gender				
Male	7 (32%)	3 (33%)	10 (48%)	20 (38%)	0.534 *
Female	15 (68%)	6 (67%)	11 (52%)	32 (62%)	
	Age (in years)				
Mean age	45.8	44.1	44.4	44.9	0.937 **
Standard deviation	12.876	18.462	13.905	14.075	
	Presence of symptoms				
No	11 (58%)	5 (71%)	13 (68%)	29 (64%)	0.728 *
Yes	8 (42%)	2 (29%)	6 (32%)	16 (36%)	
	Presence of local swelling				
No	9 (53%)	7 (100%)	9 (47%)	25 (58%)	0.047 *
Yes	8 (47%)	0	10 (53%)	18 (42%)	
	Radiological limits				
Well-defined	7 (39%)	4 (44%)	15 (75%)	26 (55%)	0.063 *
Ill-defined	11 (61%)	5 (56%)	5 (25%)	21 (45%)	

* Pearson's chi-squared;

** ANOVA

The mean size of the gross specimens was 336.71 mm^3^, larger in large PCs than in PGs and small PCs (Table 2). Most cases showed moderate/intense chronic inflammatory infiltrates, but no difference among our three groups (Table 2). We observed no significant difference in the thickness of the epithelial lining between small and large PCs (Table 2). We also found cholesterol crystals clefts in three PCs (10%).

**Table 2:** Distribution of the histological parameters by group (PG - periapical granuloma; PC - periapical cyst)

Histological parameter	Group				P-value
	PG	Small PC	Large PC	Total	
	Size of the gross specimen (in mm3)				
Mean size	161.5	115.67	628.9	336.71	0.024 *
Standard deviation	22.637	246.468	894.278	625.774	
	Intensity of the inflammatory infiltrate				
Focal/Slight	6 (27%)	4 (44%)	7 (33%)	17 (33%)	0.65 **
Moderate/Intense	16 (73%)	5 (56%)	14 (67%)	35 (67%)	
	Type of inflammatory infiltrate				
Chronic	16 (73%)	7 (78%)	20 (95%)	43 (83%)	0.136 **
Mixed (chronic + acute)	6 (27%)	2 (22%)	1 (5%)	9 (17%)	
	Thickness of the epithelium lining				
Hyperplastic	NA ***	4 (44%)	16 (76%)	20 (67%)	0.091 **
Atrophic	NA ***	5 (56%)	5 (24%)	10 (33%)	

* ANOVA;

** Pearson's chi-squared;

*** Not applicable

**Figure 1:**
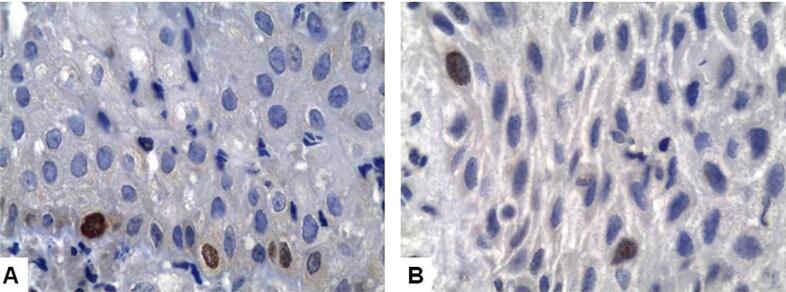
A and B - Nuclear Ki-67 expression in the epithelial lining of two periapical cysts (Immunoperoxidase, 1000x)

The mean cellular proliferation index of the epithelial lining of PCs, measured by Ki-67 expression, was 3.02 (DP±2.98), ranging from 0 to 12.6 positive nuclei per high-power field (Figure 1). Small PCs had a 2.2 (DP±1.6) mean score, whereas large PCs, a 3.4 (DP±3.4) mean score (p=0.306). PCs with focal/slight or moderate/intense inflammation had, respectively, 3.1 (DP±4.1) and 3 (DP±2.8) mean scores (p=0.951). PCs with chronic or mixed inflammatory infiltrates had, respectively, 3.1 (DP±3.1) and 2 (DP±1.7) mean scores (p=0.545). PCs with predominantly atrophic or hyperplastic epithelia had, respectively, 2.6 (DP±3.8) and 3.2 (DP±2.6) mean scores (p=0.592).

We found CD57+ cells in 54% of the IPLs, with no difference on their frequency among the groups (Table 3), and Bcl-2 expression in 65% of the IPLs, with no significant difference on its expression among the groups (Table 3). We observed EGF and KGF expression in, respectively, 54% and 58% of the IPLs, both more common in PCs than in PGs (Table 3). However, we found no statistically significant difference in the frequency of CD57, KGF, EGF, and EGFR immunopositive cells in PGs, and small and large PCs. We observed no statistically significant difference in the presence of symptoms and local swelling according to our histological parameters, the presence of CD57+ cells, and Bcl-2, EGF, and KGF expression. When comparing the frequency of CD57, KGF, EGF, and EGFR immunopositive cells to the presence of symptoms and local swelling, EGF-positive cells were more frequent in symptomatic (53.75%±23.058) than in asymptomatic (37.59±22.104) cases (p=0.03).

**Table 3:** Distribution of CD57+ cells and Bcl-2, EGF, and KGF expression in periapical granulomas (PGs, n=22) and in small (n=9) and large (n=21) periapical cysts (PCs)

Parameter	Categorization		P-value *
CD57+ cells	Absent	Present	0.748
PGs	9 (41%)	13 (59%)	
Small PCs	4 (44%)	5 (56%)	
Large PCs	11 (52%)	10 (48%)	
Bcl-2 expression	Negative/focal	Positive	0.328
PGs	10 (45%)	12 (55%)	
Small PCs	3 (33%)	6 (67%)	
Large PCs	5 (24%)	16 (76%)	
EGF expression	Negative/focal	Positive	0.0001
PGs	20 (91%)	2 (9%)	
Small PCs	1 (11%)	8 (89%)	
Large PCs	3 (14%)	18 (86%)	
KGF expression	Negative/focal	Positive	0.0001
PGs	15 (68%)	7 (32%)	
Small PCs	1 (11%)	8 (89%)	
Large PCs	6 (29%)	15 (71%)	

* Pearson's chi-squared

We found CD57+ cells more frequently in IPLs with moderate/intense inflammatory infiltrates (Table 4), and no difference in Bcl-2, EGF, and KGF expression according to the intensity of inflammatory infiltrates (Table 4). The presence of CD57+ cells and Bcl-2, EGF, and KGF expression were not significantly different between IPLs with chronic and mixed inflammatory infiltrates or between PCs with atrophic and hyperplastic epithelial lining. We observed no statistically significant difference in the presence of CD57+ cells and Bcl-2, EGF, and KGF expression according to the mean gross size of the IPLs. When comparing the frequency of CD57, KGF, EGF, and EGFR immunopositive cells to the intensity and type of inflammatory infiltrates and epithelial thickness, CD57-positive cells were more frequent in moderate/intense (9.91±8.552) versus focal/slight (5.12±3.822) inflammatory infiltrates (p=0.007).

**Table 4:** Distribution of CD57+ cells and Bcl-2, EGF, and KGF expression according to the intensity of the inflammatory infiltrate (Focal/Slight - n=17; Moderate/Intense - n=35) in the inflammatory periradicular lesions studied

Intensity of the inflammatory infiltrate	Parameter		P-value *
	CD57+ cells		0.0001
	Absent	Present	
Focal/Slight	15 (88%)	2 (12%)	
Moderate/Intense	9 (26%)	26 (74%)	
	Bcl-2 expression		0.943
	Negative/focal	Positive	
Focal/Slight	6 (35%)	11 (65%)	
Moderate/Intense	12 (34%)	23 (66%)	
	EGF expression		0.53
	Negative/focal	Positive	
Focal/Slight	6 (35%)	11 (65%)	
Moderate/Intense	4 (11%)	31 (89%)	
	KGF expression		
	Negative/focal	Positive	0.423
Focal/Slight	6 (35%)	11 (65%)	
Moderate/Intense	16 (46%)	19 (54%)	

* Pearson's chi-squared

**Figure 2:**
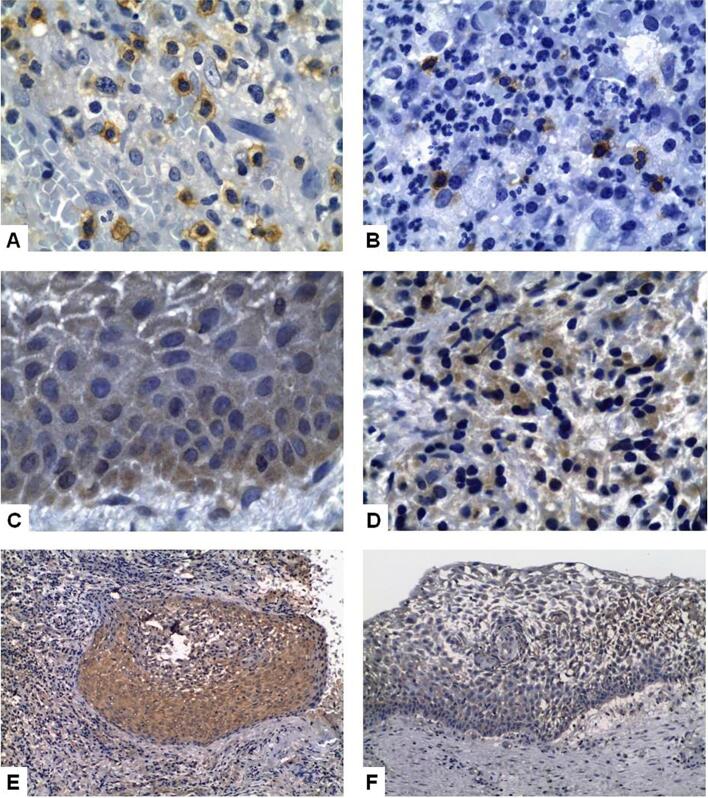
Presence of CD57+ cells in an inflammatory periradicular lesions with a chronic inflammatory infiltrate (A, Immunoperoxidase 1000x), and a mixed inflammatory infiltrate (B, Immunoperoxidase 1000x); Bcl-2 expression in the epithelium (C, Immunoperoxidase 1000x) and in the connective tissue showing chronic inflammation (D, Immunoperoxidase 1000x) in a periapical cyst; EGF expression in the epithelium of an initial periapical cyst (E, Immunoperoxidase 200x), and in the hyperplastic epithelium of an advanced periapical cyst (F, Immunoperoxidase 200x)

**Figure 3:**
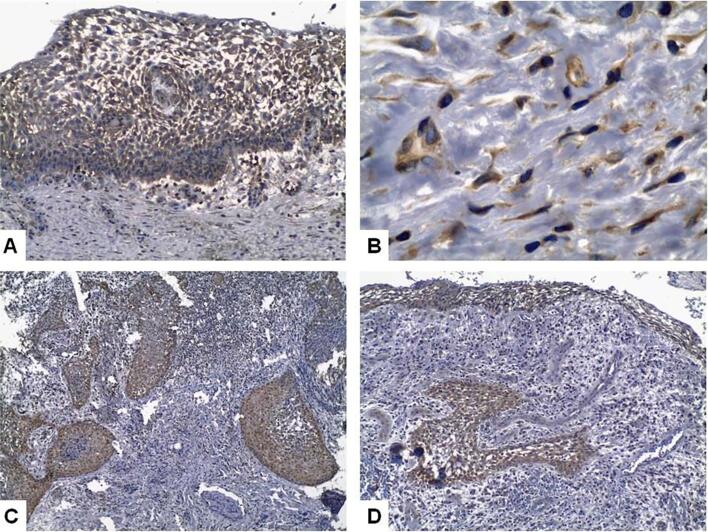
KGF expression in the hyperplastic epithelial lining (A, Immunoperoxidase 200x) and in fibroblasts in the fibrous capsule of a periapical cyst (B, Immunoperoxidase 1000x); EGFR expression in proliferating odontogenic epithelium in the capsule of a periapical cyst (C, Immunoperoxidase 100x), and in the epithelial lining and in the proliferating epithelial rest in the capsule of a periapical cyst (D, Immunoperoxidase 200x)

We evaluated EGFR expression in 40 specimens, including 22 PGs and 18 PCs; none of the latter showed EGFR expression. Among the 18 PCs, we found no statistically significant difference in EGFR expression between small and large PCs or the type and intensity of inflammatory infiltrates, and the thickness of the epithelial lining. Figures 2 and 3 show the presence of CD57+ cells and Bcl-2, EGF, KGF, and EGFR expression in the IPLs evaluated in this study.

## Discussion

The role of epithelial growth factors, apoptosis-regulating proteins, and CD57+ cells (including natural killer cells and CD8+ T cells) in the pathogenesis and development of PGs and PCs has been previously investigated.^4-7,16-18^ However, few studies evaluated the importance of these factors by comparing inflammatory periradicular lesions at different stages of development.

The CD57 protein is a marker expressed by natural killer cells, which possess a natural cytotoxic ability against tumor cells.^9^ Moreover, CD8+ T cells expressing the CD57 protein play an important role in mediating immunosuppression.^9^ In this study, we found CD57+ cells in approximately half of the lesions studied – a greater frequency than previously reported in the literature.^19^ We observed a greater presence of CD57+ cells in IPLs with moderate/intense inflammatory infiltrates, which may relate to a greater inflammatory response in general.^8^ We found no significant differences among the groups or between PCs with different epithelial linings, although previous studies have reported that PCs with atrophic epithelial lining show significantly higher numbers of CD57+ cells.^8,20^ Our results suggest that the cytotoxic activity of CD57+ cells is not associated to the proliferation of epithelial cells in the lining of PCs.

The Bcl-2 protein is an important mediator of cell division regulation. It can inhibit apoptosis and extend the survival of several types of cells, which may stimulate the development of PCs and other types of odontogenic cysts.^7,10,17,21,22^ In this study, comparing the groups showed no difference in Bcl-2 expression according to the type and intensity of inflammatory infiltrates or the type of epithelial lining in the PCs. Nonetheless, previous studies indicated that the expression of apoptosis-regulating factors may vary according to the structure of the epithelial lining and the inflammatory components present in PCs and other odontogenic cysts.^7,22,23^ Loyola, et al.^17^ (2005) showed greater Bcl-2 expression in lesions with atrophic epithelial lining than in those with hyperplastic epithelial lining, although the authors found no significant difference between the groups studied. Our results fail to support that anti-apoptotic Bcl-2 activity in mitochondrial and membrane cellular compartments relates to the survival, maintenance, and proliferation of epithelial cells in the lining of PCs.

EGF stimulates the proliferation of epithelial cells, hepatocytes, and fibroblasts via its interaction with receptors in the membrane of target cells.^5,24,25^ The literature associates this protein with various vital functions in normal and altered cells, such as migration, proliferation, mobility, and wound healing.^5,24,25^ Our results show no difference in EGF expression regarding the type and intensity of inflammatory infiltrates and the type of epithelial lining in the cystic lesions. Still, when comparing PGs and PCs, the latter showed greater EGF expression. These findings corroborate previous studies^5^ and support the hypothesis that EGF expression plays a role in stimulating epithelial cell proliferation, thus taking part in cyst formation. We should mention, however, that we found no difference in EGF expression between small and large PCs.

We normally observe EGFR expression in epithelial cells with proliferative potential.^4,16,25-27^ EGFR activation by EGF binding increases tyrosine kynase activity and stimulates signal transduction cascades, which are associated with epithelial cell proliferation.^4,16,25-27^ The literature has shown its expression in the epithelia of IPLs, especially in the lining of PCs, suggesting that EGFR expression may be important for maintaining these lesions.^4,16,28,29^ Additionally, the presence of an infection seems to modulate EGFR expression in odontogenic cysts, and may influence the proliferative ability of odontogenic epithelia.^27^ In our study, however, EGFR expression failed to show a correlation with any of the parameters evaluated, including between small and large PCs.

Fibroblasts produce KGF, a mitogen involved in epithelial cell growth and differentiation. KGF also contributes toward vital functions such as tissue repair. The presence of inflammation in some chronic oral conditions seems to influence its expression.^11,30^ In this study, KGF showed greater expression in PCs than in PGs, and in small PCs than in large PCs. These differences suggest that KGF may play a role in the mechanism of PC formation and that its effect is more significant in the initial stages of cyst development, possibly similar to its epithelial proliferative effect during wound healing.^11,30^ KGF expression was not significantly different to the intensity and type of inflammatory infiltrates, contrasting with the information reported in the literature.^6^ Epithelial cells were the cell types with greater KGF expression, but comparing atrophic and hyperplastic epithelia showed no statistical difference.

We found the antigen Ki-67 expressed in the nuclei of epithelial cells in 90% of the PCs evaluated. These values are above the 60% reported in a previous study.^18^ In the epithelial lining of PCs, Ki-67 expression was limited, in the range of 1-5%, values similar to what a previous study reports.^14^ We found no difference in the mean cell proliferation index among the groups studied; this index was similar in lesions with different types and intensities of inflammatory infiltrates and in cystic lesions with different epithelia. Still, we found greater Ki-67 expression in PCs with intense inflammatory infiltrates, suggesting that inflammations may play at least a partial role in modulating the proliferation of cystic epithelia, as previous studies have suggested.^7,23^

## Conclusions

The results from this study indicated that the more frequent EGF and KGF expression in PCs and the greater presence of CD57+ cells in lesions with intense inflammatory infiltrates suggest these factors influence the development of IPLs. Moreover, the greater KGF expression in initial PCs suggests its importance in the initial stages of PC development.
